# Effect of *Helicobacter pylori* infection on chronic periodontitis by the change of microecology and inflammation

**DOI:** 10.18632/oncotarget.11449

**Published:** 2016-08-20

**Authors:** Zhekai Hu, Yu Zhang, Zhiyu Li, Yuedi Yu, Wenyan Kang, Yingnan Han, Xiwen Geng, Shaohua Ge, Yundong Sun

**Affiliations:** ^1^ Shandong Provincial Key Laboratory of Oral Tissue Regeneration, School of Stomatology, Shandong University, Jinan, Shandong 250012, People's Republic of China; ^2^ Department of Microbiology, Key Laboratory for Experimental Teratology of Chinese Ministry of Education, School of Medicine, Shandong University, Jinan, Shandong 250012, People's Republic of China; ^3^ Shanghai Southwest Weiyu Middle School, Shanghai 200233, People's Republic of China; ^4^ Laboratory of Oral Tumor Biology, Shanghai Research Institute of Stomatology Ninth People Hospital, Shanghai Jiao Tong University School of Medicine, Shanghai 200011, People's Republic of China; ^5^ Department of Periodontology, School of Stomatology, Shandong University, Jinan, Shandong 250012, People's Republic of China

**Keywords:** Helicobacter pylori, Wnt5a, IL-8, chronic periodontitis, inflammation

## Abstract

*Helicobacter pylori (H. pylori),* a pathogen inducing peptic disease, is recently found to be binding to the progress of periodontitis. Most previous studies are case-controlled, and they investigate the risk of *H. pylori* infection in disease the development of while few studies evaluate the correlation between *H. pylori* and periodontal pathogens. Therefore, we investigated the correlation between *H. pylori* infection with periodontal parameters, periodontal pathogens and inflammation. The results indicated that patients with *H. pylori* showed significantly higher probing depth and attachment loss than those without (*p* < 0.05). Among 28 subgingival plaque samples from 14 patients, the frequencies of *Porphyromonas gingivalis, Prevotella intermedia, Fusobacterium nucleatum* and *Treponema denticola* were significantly higher with *H. pylori* infection than those without *H. pylori* infection (*p* < 0.05). However, the frequency of *Aggregatibacter actinomycetemcomitans* was lower (*p* < 0.05). Furthermore, after human acute monocytic leukemia cell line (THP-1) was stimulated with *cagA*-positive standard strains (*cagA^+^ H. pylori* 26695), the expression of periodontitis-related molecules Wnt5a, interleukin 8 (IL-8), interleukin 6 (IL-6) and interferon gamma (IFN-γ) significantly increased (*p* < 0.05). Conversely, the expression of tumor necrosis factor alpha (TNF-α) was almost stable. Meanwhile, *cagA^+^ H. pylori* promoted significantly higher expression of IL-8 and Wnt5a than isogenic *cagA* mutants strains (*cagA^−^ H. pylori* 26695) did. Taken together, our data suggested that *H. pylori* might promote the growth of some periodontal pathogens and aggravate the progress of chronic periodontitis.

## INTRODUCTION

Periodontitis is one of the most prevalent diseases, and it influences up to 90% people in the world [1]. Varying from other infectious diseases, periodontitis is a multiple bacteria-related disease. As the most important etiology, periodontal pathogens have been studied for decades. In 1996, 11 periodontitis-related pathogens had been determined including three strong associated pathogens and eight moderate associated pathogens including *Aggregatibacter actinomycetemcomitans, Porphyromonas gingivalis, Bcteroides forsythus*, *Prevotella intermedia, Prevotella nigrescens,* etc [2].

Multiple experimental methods have been used in detecting periodontal pathogens. However, more than 50% of bacteria cannot be cultivated. In recent years, techniques such as PCR, sequencing and DNA hybridization have been used to identify bacterial species in samples such as plaque and soil [3], and about 1,000 bacterial species have been found in human oral cavity [4], which led to a development in the area of periodontitis as well. Previous studies found that periodontal diseases occurred due to the change of periodontitis-related bacterial species in subgingival plaque [5]. Meanwhile, the correlation between oral diseases and systematic diseases such as rheumatic heart disease, glomerulonephritis and gastric disease has been reported these years [6–8]. Among these, the effect of *Helicobacter pylori* (*H. pylori*) on periodontitis became a new hotspot.

*H. pylori* is a typical Gram-negative organism which needs to grow under a microaerophilic environment. It is generally studied for its peptic disease-induced function [9]. With the development of the detection methods, *H. pylori* has been found to have a close connection with many diseases such as chronic liver disease, oral lichen planus and periodontitis [10]. Desai et al. first found the bacteria in human plaque [11]. Since then, the connection between *H. pylori* and periodontal diseases had been studied. Previous reports have found that people with *H. pylori* infection tend to have periodontitis.

Meanwhile, considerable research articles including clinical and experimental studies have reported the relationship between *H. pylori* infection and periodontitis [12–14]. However, some studies showed that there was no correlation between *H. pylori* infection and periodontal status [15]. Moreover, most the related studies focus on clinical index, few investigated the correlation between microorganisms and the molecular expression of inflammatory proteins. Those seemed to be apparent defects in this research area. What we have done was to detect the correlation among those by the methods of molecular biotechnology. The results did show us some interesting phenomenon.

Moreover, latest study reported the Wingless proteins (Wnt) family and the expression of cytokines were correlated to the process of chronic periodontitis [16]. Wnt is one of the most important proteins involved in the development of embryo and the differentiation of blood cells and lymphocytes [17]. Among those, Wnt5a came into our view. Wnt5a could be secreted by inflammatory tissues, antigen-presenting cells and rheumatoid arthritis joints [18] and promote tumor proliferation and stromal vascular endothelial growth factor [19]. In primary human gingival fibroblasts (HGF), the expression of Wnt5a mRNA was rather constant after stimulation with *P. gingivalis* LPS. On the contrary, in primary monocytes, the expression of Wnt5a mRNA significantly increased by *P. gingivalis* LPS and reduced by using NF-kB inhibitor MG132. These results suggest that monocytes, but not HGF, play an important role in Wnt5a up-regulation at inflamed site. In this study, Wnt5a served as an important molecule to detect the status of inflammation.

Cytokines could induce inflammation and act as important molecules in angiogenesis [20], the progress of human chronic periodontal inflammation and the development of some cancer [21–23]. Cytokines such as interleukin 6 (IL-6), interleukin 8 (IL-8), tumor necrosis factor alpha (TNF-α) and interferon gamma (IFN-γ) could be produced by immunocytes in inflammatory tissues. Numerous studies examining protein expression in patients with periodontitis also showed that periodontitis would increase the secretion of those cytokines [24, 25]. Cytokine played an important role in the progression of chronic periodontitis as well [26]. Meanwhile, many reports found that *H. pylori* infection associated with a *cagA*-positive phenotype could stimulate the immune system and gastric epithelial cell lines, which would lead to the release of cytokines [27]. Reti KL found dysbacteriosis could also happen by the change of IL-8 concentration [28]. All of those suggested that cytokines were important in the progress of *H. pylori* infection.

Hence, in this study, we aimed to determine the association of *H. pylori* infection with periodontal disease, and the difference in microorganism environment with and without the infection and the effect of *H. pylori* on immune cells.

## RESULTS

### Detection of *H. pylori* infection by real-time PCR

28 samples from 14 patients with chronic periodontitis were involved in our study, and real-time PCR was performed to determine if they were infected with *H. pylori*. All basic clinical features of two groups were shown in Table 1 and Table 2. By statistical analysis, no significant difference in basic periodontal clinical characteristics which suggested the experiment grouping design was appropriate (Table 1). The result indicated that patients with and without *H. pylori* infection did not differ in plaque index (PLI) and bleeding index (BI) (*p* > 0.05) (Table 2), while patients with *H. pylori* exhibited deeper probing depth (PD) and more attachment loss (AL) than those without (*p* < 0.05) (Table 2).

**Table 1:** Basic periodontal clinical characteristics of patients with and without *Helicobacter pylori* infection

Characteristics	*H. pylori* positive	*H. pylori* negative
Age (years; mean ± SD)	35.15 ± 2.891	38.00 ± 2.580
Males	9 (13)	8 (15)
Smokers	0	0
Antibiotic usage	0	0
Systemic diseases		
Cardiovascular disease	0	0
Diabetes	0	0
Arthritis	0	0
Thyroid	0	0
Asthma	0	0
Depression	0	0

No difference between groups by *t* test.

**Table 2:** Periodontal indexes of patients with and without *H. pylori* infection

Periodontal indexes	*H. pylori* positive	*H. pylori* negative	*p* value
Plaque index	2.231 ± 0.1216	2.067 ± 0.0667	*p* > 0.05
Bleeding index	2.462 ± 0.2683	2.333 ± 0.1869	*p* > 0.05
Probing depth (mm)	5.846 ± 0.4507*	4.667 ± 0.1869	*p* < 0.05
Attachment loss (mm)	7.231 ± 0.3782*	6.133 ± 0.2153	*p* < 0.05

Data were expressed as mean ± SD. Periodontal index of each site from selected patients were measured.

* There was significant difference between patients with and without *H. pylori* in probing depth and attachment loss.

### Bacteria in plaque samples

**Figure 1:**
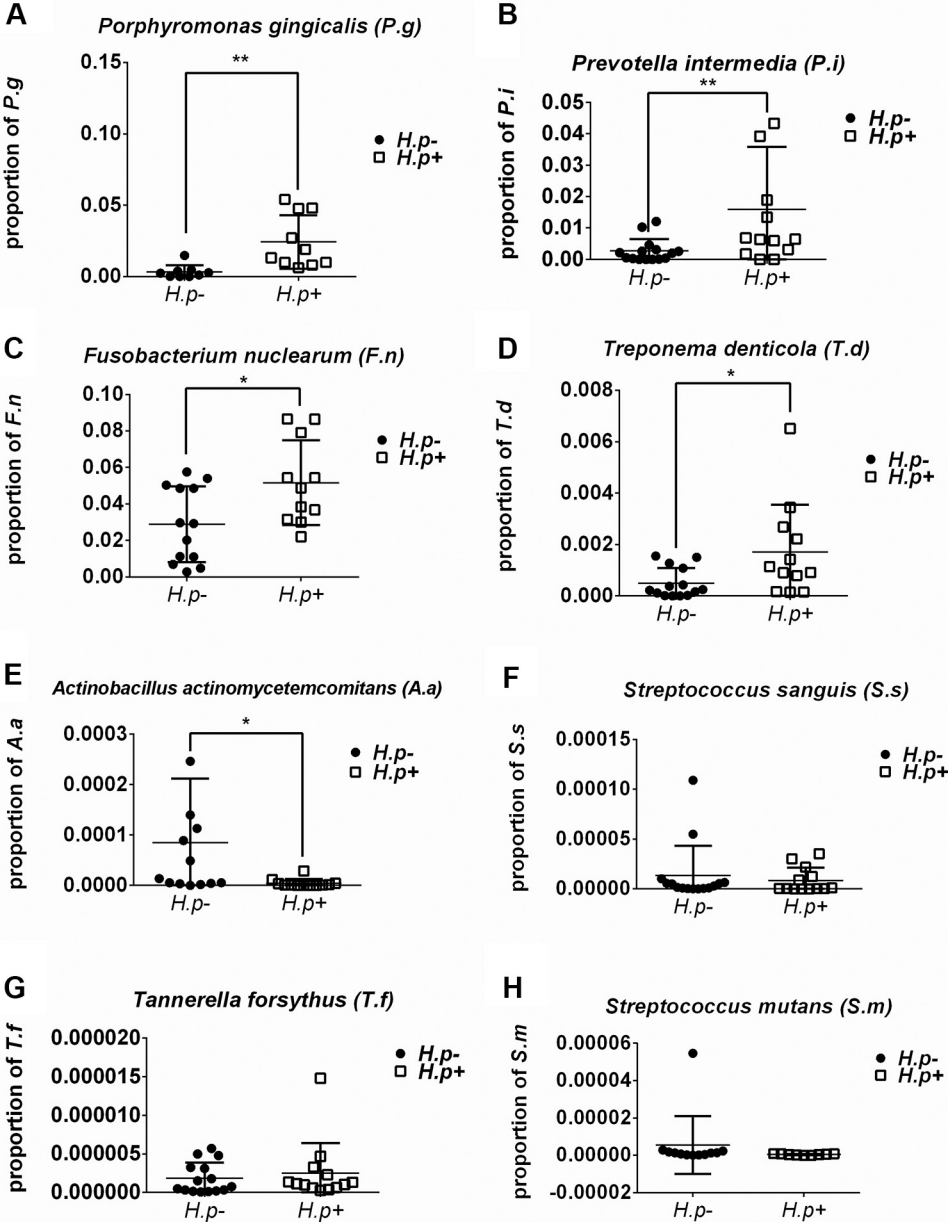
Frequency of bacteria in *H. pylori*-positive and -negative subgingival plaque samples Real-time PCR detection of target bacteria including *Porphyromonas gingivalis* (**A**), *Prevotella intermedia* (**B**), *Fusobacterium nuclearum* (**C**), *Treponema denticola* (**D**), *Aggregatibacter actinomycetemcomitans* (**E**), *Streptococcus sanguis* (**F**), *Tannerella forsythus* (**G**) and *Streptococcus mutans* (**H**). The proportion of target bacterium was calculated by comparing the circulation threshold of target bacterium to that of the universal primer. *p < 0.05; **p < 0.01 by *t*-test.

**Figure 2:**
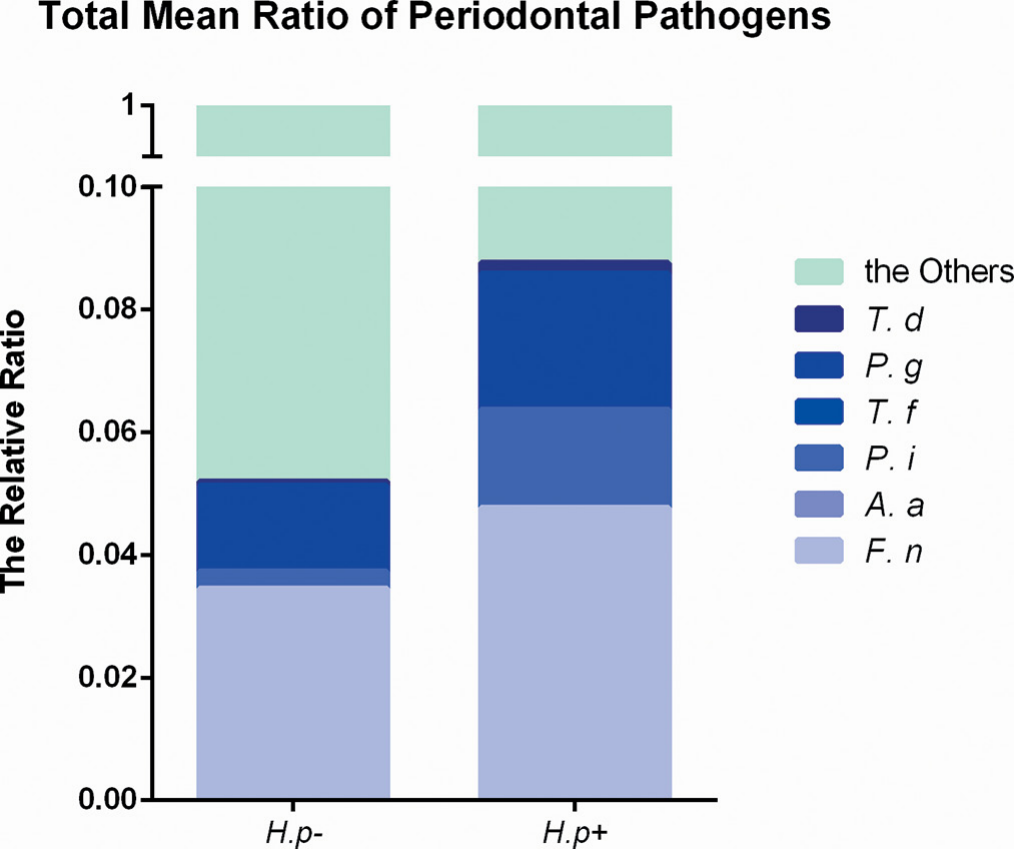
Total ratio of periodontal pathogens in *H. pylori*-positive and -negative subgingival plaque samples Real-time PCR detection of periodontal pathogens including *Porphyromonas gingivalis*, *Prevotella intermedia*, *Fusobacterium nuclearum*, *Treponema denticola*, *Aggregatibacter actinomycetemcomitans* and *Tannerella forsythus*. The proportion of target bacteria was calculated by comparing the circulation threshold of target bacteria to that of the universal primer. The total ratio of periodontal pathogens was added by calculating the mean of each bacterium.

The frequencies of different bacteria in subgingival plaque samples from patients with or without *H. pylori* infection (Figure 1) were determined. By two-way *t*-test, the frequencies of *S. mutans* and *S. sanguis* showed no significant relation with the infection of *H. pylori.* However, those of *P. gingivalis*, *P. intermedia*, *F. nuclearum* and *T. denticola* were significantly higher in samples with *H. pylori* infection than those without (*p* < 0.05). Conversely, the frequency of *A.actinomycetemcomitans* was lower in *H. pylori* infection samples (*p* < 0.05). Therefore, not all bacteria in subgingival plaque had a close relationship with *H. pylori*. And the total ratio of periodontal pathogens in *H. pylori*-positive samples was more than that in *H. pylori*-negative samples (Figure 2).

### The expression of Wnt5a increased in THP-1 cells with *c*agA^+^ *H. pylori* and *cagA*^−^ *H. pylori* stimulation

**Figure 3:**
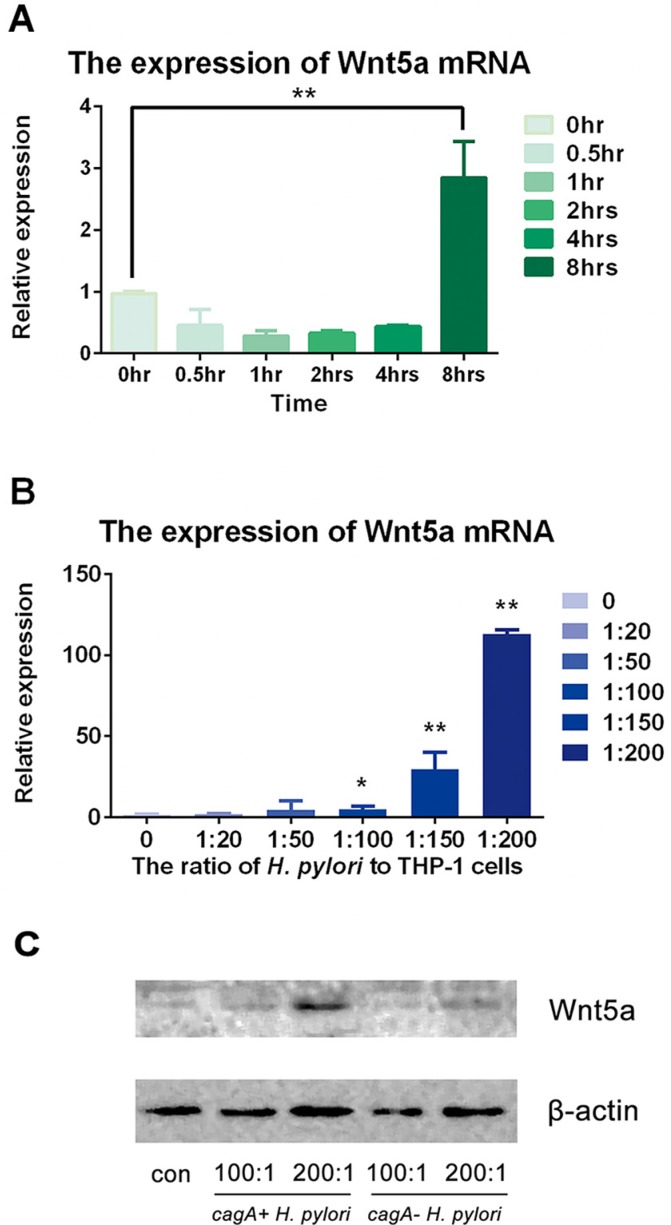
*H. pylori* up-regulated expression of Wnt5a in mRNA and protein levels (**A**) *H. pylori* was used to infect THP-1 cells at various times at a ratio of 100:1. Real-time PCR was used to detect the expression of Wnt5a mRNA compared to that of GAPDH. (**B**) *H. pylori* was used to infect the THP-1 cells for 8 hrs at ratios of 10:1, 20:1, 50:1, 100:1, 150:1 and 200:1. (**C**) THP-1 cells were stimulated by *cagA*+ *H. pylori* and *cagA*- *H. pylori* for 8 hrs at ratios of 0, 100:1 and 200:1. Cells were extracted, and total protein was obtained. Western blot was used to detect protein by antibody against Wnt5a, and β-actin was used as the protein control. Real-time PCR was used to detect the expression of Wnt5a mRNA. Data were expressed as mean ± SD from 3 experiments. *p < 0.05; **p < 0.01 by one-way ANOVA.

THP-1 cells were infected with *cagA*^+^ *H. pylori* for different time points (0, 0.5, 1, 2, 4 and 8 hrs) and at different ratios (0, 20:1, 50:1, 100:1, 150:1 and 200:1). Then, mRNA and total protein were obtained to detect the expression of Wnt5a in mRNA and protein level. Our results illustrated that the expressions of Wnt5a mRNA in THP-1 cells stimulated with *H. pylori* were stable at 0, 0.5, 1, 2 and 4 hrs (*p* > 0.05) while increased dramatically at 8 hrs (*p* < 0.05) (Figure 3A). Moreover, *H. pylori* enhanced Wnt5a mRNA expression at a dose-dependent manner, with the maximum effect appeared at the ratio of 200:1 (*p* < 0.05) (Figure 3B).

Then, THP-1 cells were infected by *cagA*^+^ *H. pylori* and *cagA*^−^ *H. pylori* at increasing ratio of *H. pylori* (at the ratios of 100:1 and 200:1) for 8 hrs. Wnt5a expression increased in both groups. However, more dramatic expression happened in the group of *cagA*^+^ *H. pylori* (Figure 3C).

### The expression of IL-8 increased in THP-1 cells with stimulation of *H. pylori*

**Figure 4:**
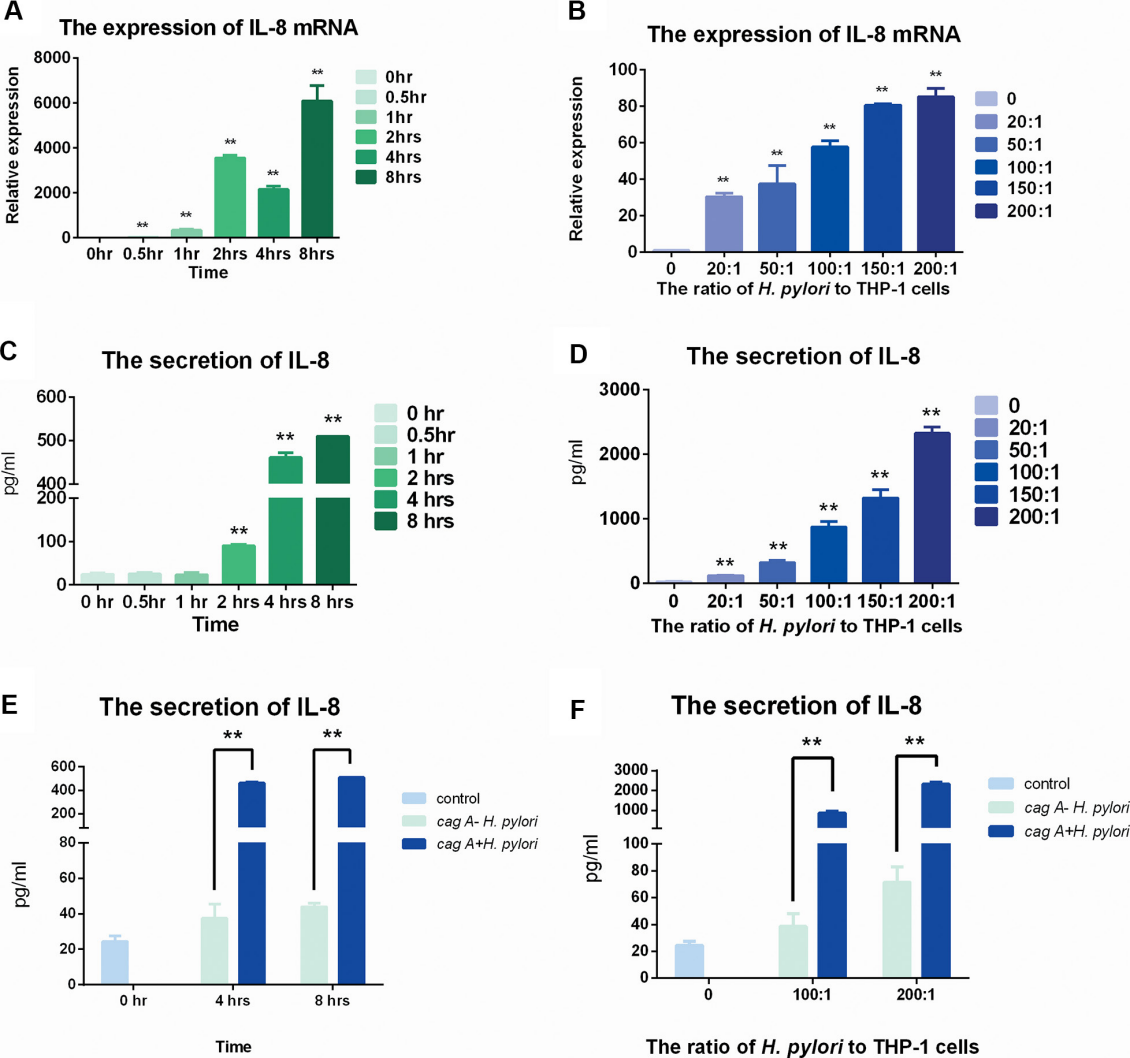
The expression of IL-8 in mRNA and protein levels after stimulation of *cagA^+^ H. pylori* and *cagA*^−^ *H. pylori.* (**A**) *H. pylori* was used to infect THP-1 cells for various times at a ratio of 100:1. Real-time PCR was used to detect the expression of IL-8 mRNA. (**B**) *H. pylori* was used to infect THP-1 cells for 8 hrs at ratios of 0, 10:1, 20:1, 50:1, 100:1, 150:1 and 200:1. Real-time PCR was used to detect the expression of IL-8 mRNA. (**C**) *H. pylori* was used to infect the THP-1 cells for various times at a ratio of 100:1. ELISA was used to detect the secretion of IL-8. (**D**) *H. pylori* was used to infect THP-1 cells for 8 hrs at ratios of 0, 10:1, 20:1, 50:1, 100:1, 150:1 and 200:1. ELISA was used to detect the secretion of IL-8. Data were expressed as mean ± SD from 3 experiments. (**E**) *cagA*^+^ *H. pylori* and *cagA*^−^ *H. pylori* were used to infect the THP-1 cells for 4 hrs and 8 hrs at a ratio of 100:1. (**F**) *cagA*^+^ *H. pylori* and *cagA*^−^ *H. pylori* were used to infect the THP-1 cells for 8 hrs at ratios of 0, 100:1 and 200:1. Data were expressed as mean ± SD from 3 experiments. *p < 0.05; **p < 0.01 by one-way ANOVA.

RT-PCR and ELISA were used to evaluate the expression of IL-8. The expression and secretion of IL-8 showed time- and dose-dependent manners (Figure 4).

Interestingly, in the protein secretion level, there were no differences among 0 hr, 0.5 hr or 1 hr. And significant differences happened from 2 hrs to 8 hrs, and similarly peaked at 8 hrs (*p* < 0.05) (Figure 4C).

In dose-control groups, the expression of IL-8 gradually increased in mRNA level, and peaked at the ratio of 150:1 while stable increase could be found in protein secretion level (*p* < 0.05) (Figure 4B, Figure 4D).

After stimulated by *cagA*^−^ *H. pylori*, the expression of IL-8 increased slowly, but was still far down from the stimulation by *cagA*^+^ *H. pylori* in both dose and time control groups (Figure 4E, Figure 4F).

### The secretions of IL-6 and IFN-γ increased but that of TNF-α was stable in THP-1 cells with stimulation of *H. pylori*

**Figure 5:**
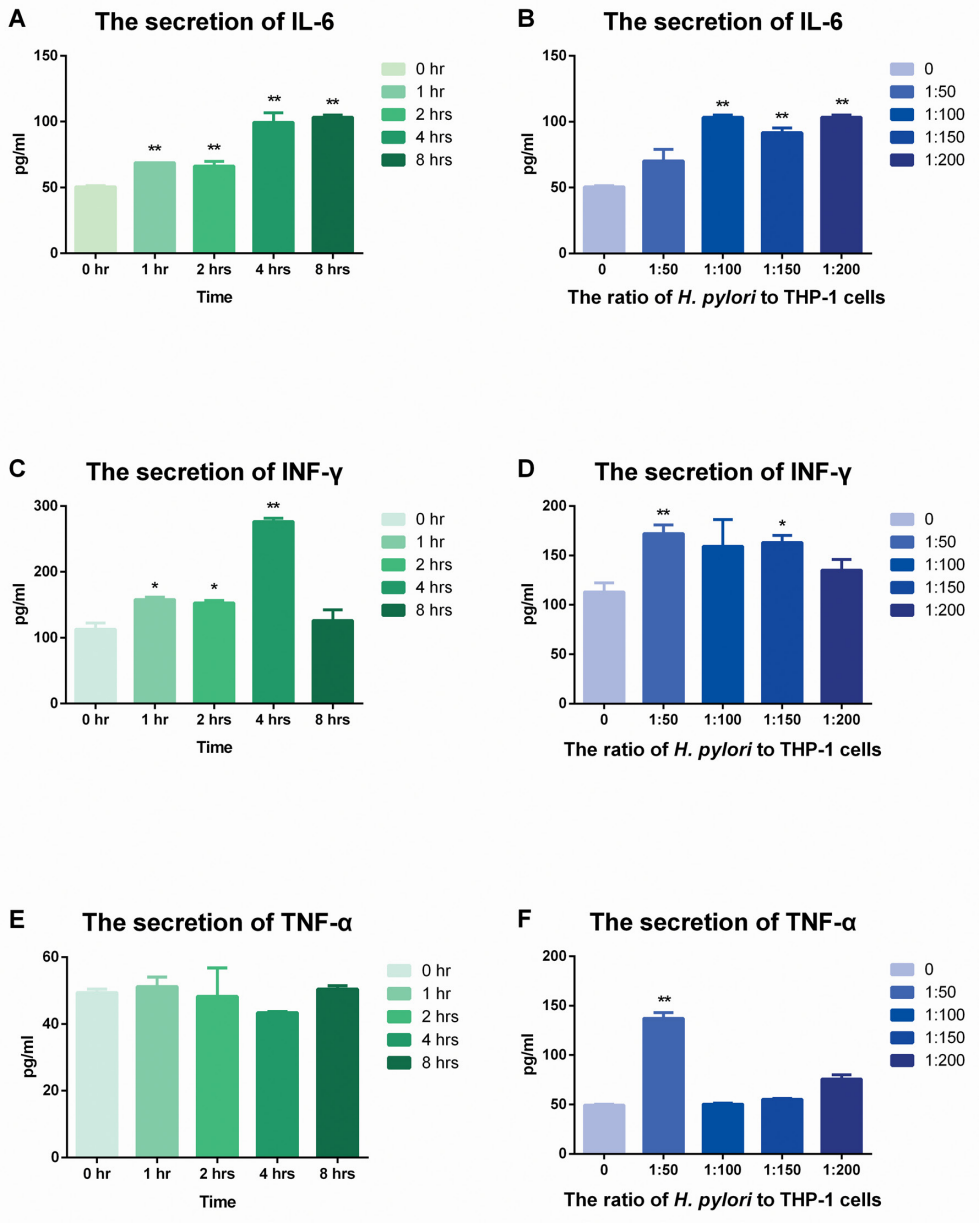
The secretion of IL-6, TNF-α and IFN-γ after stimulation of *H. pylori.* (**A**) (**C**) (**E**) *H. pylori* was used to infect THP-1 cells for various times at a ratio of 100:1. ELISA was used to detect the secretion of IL-6, TNF-α and IFN-γ. (**B**) (**D**) (**F**) *H. pylori* was used to infect THP-1 cells for 8 hrs at ratios of 0, 50:1, 100:1, 150:1 and 200:1. ELISA was used to detect the secretion of IL-6, TNF-α and IFN-γ. Data were expressed as mean ± SD from 3 experiments. *p < 0.05; **p < 0.01 by one-way ANOVA.

ELISA was used to evaluate the expression of IL-6, IFN-γ and TNF-α. The secretion of these three cytokines showed different manners from IL-8 and the secretion quantities of those were much lower than that of IL-8 (Figure 5).

Firstly, IL-6 showed time- and dose-dependent manners in protein levels (Figure 5A, Figure 5B). Likewise, the secretion of IFN-γ was increased from 0 hr to 4 hrs, but declined afterwards. While in dose control group, the highest expression occurred in the ratio of 50:1 but fell down since then.

Conversely, the secretion of TNF-α was relatively stable after stimulation with *H. pylori*. Rare dramatic changes could be found in both dose and time control groups.

## DISCUSSION

In this study, clinical features were analyzed and deeper PD and AL were found in sites with *H. pylori* infection. Then in plaque samples, we found the frequencies of *S. mutans* and *S. sanguis* showed no correlation with the infection of *H. pylori*. However, those of *P. gingivalis*, *P. intermedia*, *F. nuclearum* and *T. denticola* were significantly higher in samples with *H. pylori* infection than those without. Conversely, the frequency of *A.actinomycetemcomitans* was lower in *H. pylori* infection samples. Meanwhile, periodontitis related protein Wnt5a and cytokines were significantly higher after stimulated with *H. pylori*.

Periodontitis is a widespread plaque-induced disease. In recent years, clinical studies found *H. pylori* in oral cavity, and considerable studies found that the severer the periodontitis was, the higher positive rate of *H. pylori* could be found [12, 29, 30]. An epidemiological study performed in the USA also reported the relationship between periodontal disease and *H. pylori* infection [13]. Although there were a few studies reported negative findings [15], most related findings were consistent with our findings. In this study, samples were collected from patients with chronic periodontitis and deeper PD and AL were found in *H. pylori*-positive sites. We supposed that *H. pylori* could aggravate periodontal destruction in patients with periodontitis. Therefore, we would like to study the concrete mechanisms under this phenomena.

A wide range of bacteria related to periodontitis was found with PCR technique. With increasing frequencies of some Gram-negative and anaerobic bacteria in biofilms, the inflammation of periodontium could deteriorate [31, 32] and cytokines secretion may increase. *P. gingivalis*, *T. forsythia*, *T. denticola*, *A. actinomycetemcomitans*, even some herpes viruses [33], HIV and some fungi [1, 34] were detected from infected periodontal tissues. Here, we determined the frequencies of 5 periodontal pathogens and 2 caries related pathogens in dental plaques with and without *H. pylori* infection by real-time PCR. We found that the frequencies of *P. gingivalis*, *P. intermedia*, *F. nuclearum* and *T. denticola* were higher in samples with *H. pylori* infection than that without, with the exception of *A.actinomycetemcomitans*. And the total ratio of periodontal pathogens was relatively higher in *H. pylori*-positive samples than those in *H. pylori*-negative samples, which suggested that *H. pylori* might aggregate chronic periodontitis by change of the subgingival microbiota.

Moreover, the relationship of *H. pylori* infection and caries-associated pathogens was also investigated. In a previous study, no correlation was found between the presence of caries-associated pathogens and *H. pylori* infection [35]. Similarly, we found that no significant correlation between caries-related bacteria and *H. pylori* infection, which suggests that *H. pylori* may be not a kind of caries-related microorganism in human subgingival microecology. Aggressive periodontitis is a relative rare disease and has different disease-related pathogens from chronic periodontitis. *A. actinomycetemcomitans* was found to be one of the most frequent pathogens identified in patients with localized aggressive periodontitis [36, 37]. In contrast, we found that the frequency of *A. actinomycetemcomitans* was lower in *H. pylori*–positive samples. Those phenomenon are very interesting, and the mechanism underneath needs further study to address.

Additionally, the level of Wnt5a mRNA expression was also investigated. It had been known that Wnt5a could be expressed by some immune cells including almost all antigen-presenting cells [38] and it has been considered as one of the key molecular in chronic periodontitis recently. In a previous study, HGF-1 cells constantly expressed Wnt5a mRNA with the stimulation of *P. gingivalis*, while the expression of Wnt5a mRNA in THP-1 cells showed a dose-dependent manner. Therefore, Wnt5a might be a periodontitis-related protein and could suggest the potential periodontal destruction [16]. In this study, THP-1 cells stimulated with *H. pylori* showed a dose- and time-increased expression and secretion of Wnt5a. Interestingly, *cag A*^+^ *H. pylori* significantly up-regulated the expression of Wnt5a. The result suggested that in response to periodontitis, the genes of Wnt superfamily may be activated by *H. pylori* and *H. pylori* may enhance the expression of Wnt5a and induce the development of periodontitis. Meanwhile, CagA might play an essential role in this process.

Furthermore, the dose- and time-dependent of IL-8 on expression were first studied. According to the result, the expressions of IL-8 increased significantly after the stimulation of *H. pylori*. The overall effect of the stimulation of *H. pylori* was consistent with previous findings [39]. The production of different cytokines could be induced by the infiltration of immunocyte (such as T lymphocytes, monocyte and neutrophils) into diseased tissues [40–42]. A previous study found that *H. pylori* mainly activated the expression of IL-8 in gastric tissue by the expression of CagA [43]. As shown in the results, the key toxic protein of CagA which is binding to stomach cancer [44] determined to be the crucial factor in those processes. Moreover, dysbacteriosis could happen by the change of IL-8 concentration [28]. TNF-α, IL-6 and IFN-γ were determined to be correlated with the severity of tissue inflammation [45–47]. Our findings confirmed that the secretion of IL-6 and IFN-γ in THP-1 cells could increase but that of TNF-α was stable after stimulation. The secretion quantities of those three cytokines were relatively lower in comparison to that of IL-8, which suggested that IL-8 was the major cytokine stimulated by *H. pylori*. Those findings might suggest that *H. pylori* could induce the response of the immune system with the invaded pathogens and the progress of inflammation could be exacerbated. It might give us a hint that cytokines might serve as mediators on dysbacteriosis.

In a word, this study shows the positive association between *H. pylori* infection and periodontal pathogens. Also, the results indicate that *H. pylori* infection could influence the chronic periodontitis by the change of microecology and inflammation, and induce the severe progress of this disease. Meanwhile, *cagA*^+^ *H. pylori* shows the similar inflammatory effect on THP-1 as *P. gingivalis* which suggests that *H. pylori* has the pathogenic effect during the development of periodontitis, CagA might play an irreplaceable role in this process. What we found might give some hints to further therapy of periodontitis, the eradication of *H. pylori* could be a key point of chronic periodontal treatment. However, the concrete mechanisms are not clear now, and more patients should be involved and further studies should be done to explore the intrinsic mechanism in future.

## MATERIALS AND METHODS

### Patient sampling

28 samples from 14 patients with chronic periodontitis from Department of Periodontology, School of Stomatology, Shandong University were collected. The subjects were informed about the procedures and aims of this experiment and signed a consent form. The study was approved by the Medical Ethical Committee of School of Stomatology, Shandong University (Protocol Number: 201302070).

A diagnosis of chronic periodontitis was determined based on the American Academy of Periodontology of Periodontology parameters [48]. Inclusion criteria were age 18 to 60 years, more than 30% sites with probing depth (PD) deeper than 4 mm; more than 30% sites with attachment loss (AL) of 2 mm, at least 2 molars remained in oral cavity. The exclusion criteria were antibiotic use in the previous 3 months, professional periodontal therapy in the previous year, diagnosis of systemic diseases that would activate periodontitis, and smoking > 10 cigarettes a day.

Periodontal index including plaque index (PLI), bleeding index (BI), probing depth (PD) and attachment loss (AL) were evaluated. With a sterilized curettage, subgingival plaque was taken from mesio-buccal site of four first molars with initial probing depth > 5 mm, and then placed in 1 ml phosphate buffered saline (PBS). After centrifuged at 3000 rpm for 5 min, the supernatants were frozen at −80°C for further processing.

### Extraction of bacterial DNA from dental plaque and real-time PCR detection of *H. pylori* infection and bacterial composition

DNA was extracted from samples and purified with the QIAamp DNAMini Kit (QIAGEN GmbH, Hilden, Germany) according to manufacturer's instructions. The purity and yield of DNA were measured by a bio-photometer (Eppendorf AG, Hamburg, Germany) with ratio from 1.6 to 1.8.

Then, Real-time PCR was carried out using 10 μl SYBR Premix Ex Taq II (Takara, Kusatsu, Japan), 2 μl sample DNA, 7.2 μl double distilled water, former primer 0.4 μl and reverse primer 0.4 μl. The primers for key bacteria investigated listed in Table 3. The primers [49] were based on 16s recombinant DNA. The primer of *H. pylori* targeted Urea A gene. We used a real-time PCR system (Bio-Rad Laboratories, USA) with the cycles pre-denaturation at 94°C for 1 min; 45 amplification cycles including denaturation at 95°C for 20 s, annealing at 60°C for 20 s, extension 72°C for 30 s; then extension at 72°C for 1 min. The relative quantification was calculated by 2^CT(key pathogen)-CT(universal primer)^.

**Table 3:** Primers for real-time PCR to detect target pathogens

Primers	Product size (bp)	Target gene	Annealing temperature (°C)
**Universal primer**			
5′-ACTCCTACGGGAGGCAGCAGT-3′	180	16s rRNA	60
5‘-TATTACCGCGGCTGCTGGC-3′			
***H. pylori***			
5′-CAAGAAGGGCGCACTCTTTT-3′	250	Ure A	60
5′-CGATTTGAACCGGTCTGTCG-3′			
***F. nuclearum***			
5′-TACCAACAGAAGAAGTGACGGCTAA-3′	162	16s rRNA	60
5′-ATACAGTTTCCAACGCAATACAGAG-3′			
***S. mutans***			
5′-GGTCAGGAAAGTCTGGAGTAAAAGGCTA-3′	260	16s rRNA	60
5′-GCGTTAGCTCCGGCACTAAGCC-3′			
***S. sanguis***			
5′-TTCAAAGCCAAGACCAAGCTAGT-3′	88	16s rRNA	60
5′-CCAGCCTGAGATTCAGCTTGT-3′			
***A. actinomycetemcomitans***			
5′-CTTACCTACTCTTGACATCCGAA-3′	77	16s rRNA	60
5′-ATGCAGCACCTGTCTCAAAGG-3′			
***P. gingivalis***			
5′-TGTAGATGACTGATGGTGAAAACC-3′	178	16s rRNA	60
5′-ACGTCATCCACACCTTCCTC-3′			
***T. forsythia***			
5′-AGCGATGGTAGCAATACCTGTC-3′	88	16s rRNA	60
5′-TTCGCCGGGTTATCCTC-3′			
***P. intermedia***			
5′-TCCACCGATGAATCTTTGGTC-3′	98	16s rRNA	60
5′-ATCCAACCTTCCCTCCACTC-3′			
***T. denticola***			
5′-AGAGCAAGCTCTCCCTTACCGT-3′	105	16s rRNA	60
5′-TAAGGGCGGCTTGAAATAATGA-3′			

### Cell culture

The human leukemia monocytic cell line (THP-1) was acquired from the biochemistry laboratory, Shandong University. The culture medium included RPMI 1640 (Gibco, New York, USA) and fetal bovine serum (Gibco, New York, USA) at 10% in total and cells were grown at 37°C with 5% CO_2_ in a suitable incubator.

### Culture of *H. pylori* and cell infection

The *H. Pylori* strains including *cagA*-positive standard strains (*cagA*^+^ *H. pylori* 26695) and isogenic *cagA* mutants (*cagA*^−^ *H. pylori* 26695) were obtained from the microbiology laboratory, Shandong University. These strains were cultured under anaerobic conditions at 37°C and on specific Brucella agar plates with 7% horse serum. The *cagA*^+^ *H. pylori* was used to stimulate cells at a ratio of 100:1 for 0, 0.5, 1, 2, 4 and 8 hrs. Then, *H. pylori* was used to stimulate cells at a ratio of 0, 20:1, 50:1, 100:1, 150:1 and 200:1 for 8 hrs. The *cagA*^−^ *H. pylori* was used to stimulate cells at a ratio of 100:1 and 200:1 for 8 hrs. The quantity of cells was calculated by cell counting and the quantity of bacteria was analyzed by spectrophotometry.

### Total RNA extraction and PCR

TRIzol (Takara, Kusatsu, Japan) was used to separate total RNA, and the quality was analyzed by bio-photometry (Eppendorf AG, Hamburg, Germany) with a ratio (260/280 nm) from 1.8–2.0. PrimeScript RT reagent Kit (Takara, Kusatsu, Japan) was used to purify the mRNA and synthesize the cDNA with a thermal cycler. Real-time PCR was conducted using SYBR Premix Ex Taq II (Takara, Kusatsu, Japan) with primers targeting human IL-8, Wnt5a and GAPDH (Table 4). The results were analyzed by 2^ΔΔCT^ method.

**Table 4:** Primers for real-time PCR to detect the expression of target genes

Primers	Product size (bp)	Target gene	Annealing temperature (°C)
**Wnt5a**			
5′-TCCTCCGTGTTGTGATGTGA-3′	225	Wnt5a mRNA	60
5′-GATACGCTGCAACACCTCTG-3′			
**GAPDH**			
5′-ACCACAGTCCATGCCATCAC-3′	226	GAPDH mRNA	60
5′-ACCACCCTGTTGCTGTA-3′			
**IL-8**			
5′-TCAGAGACAGCAGAGCACAC-3′	159	IL-8 mRNA	60
5′-GGCAAAACTGCACCTTCACA-3′			

### Western blotting

Cells were collected from the above experiments and washed twice with PBS. Total Protein Extraction Kit (Bestbio Inc. Shanghai, China) with protease inhibitor and phosphatase inhibitor was used to lyse target cells for total protein extraction. The SDS-PAGE gels (10% acrylamide) were used to resolve the exact amounts of protein, and then transferred and immobilized onto nitrocellulose membranes. The membranes were firstly incubated with Wnt5a polyclonal antibody (Proteintech Group, Inc. Chicago, USA) and then β-actin antibody (Cell Signaling Technology, Inc. Danvers, USA) at 4°C overnight. The membrane was then incubated at room temperature for an additional 60 min with secondary antibody. Finally, Immobilon™ Western Chemiluminescent HRP Substrate (Millipore Corporation, Billerica, USA) was used to detect the immune-active signal of target protein.

### Detection of human IL-8, IL-6, TNF-α and IFN-γ

THP-1 cells were used in this part to detect the secretion of IL-8, IL-6, TNF-α and IFN-γ. The procedure of stimulation was the same as culture of *H. pylori* and cell infection above. Cell supernatant, however, was collected to detect the protein. The secretion of IL-8, IL-6, TNF-α and IFN-γ were detected by ELISA kits (Ebioscience, San Diego, USA). The preparation and protocol were followed according to the instructions. In each group, the absorbance of IL-8, IL-6, TNF-α and IFN-γ was detected by spectrophotometry at 450 nm, and each experiment was repeated three times. The resulting values were expressed in pg/ml.

### Statistical analysis

Data were shown as means with standard error (SD) and then analyzed by use of SPSS Client 21.0. Variance between two groups was compared by two-way *t*-test and variance among more than two groups was evaluated by one-way ANOVA. Statistical probability of *p* < 0.05 was considered significant.
